# Biochemical mechanism of clinical resistance to rilpivirine

**DOI:** 10.1186/1471-2334-12-S1-P94

**Published:** 2012-05-04

**Authors:** Kamalendra Singh, Devendra K Rai, Bechan Sharma, Eleftherios Michailidis, Emily M Ryan, Kayla B Matzek, Maxwell D Leslie, Ariel N Hagedorn, Hong-Tao Xu, Mark A Wainberg, Bruno Marchand, Stefan G Sarafianos

**Affiliations:** 1 Christopher Bond Life Sciences Center, USA; 2 Department of Molecular Microbiology & Immunology, University of Missouri, School of Medicine, Columbia, MO, USA; 3 Department of Biochemistry, University of Allahabad, Allahabad, India; 4 McGill University AIDS Centre, Lady Davis Institute for Medical Research, Jewish General Hospital, Canada; 5 McGill University, Montreal, Quebec, Canada; 6 Department of Biochemistry, University of Missouri, Columbia, MO 65211, USA

## Background

The introduction of HAART has significantly prolonged the life span of HIV-infected patients. However, the error-prone nature of HIV-1 reverse transcriptase (HIV-1 RT) results in the emergence of drug-resistant viruses and threatens the effectiveness of HAART. HIV-1 RT is a primary target of two classes of drugs: nucleoside reverse transcriptase inhibitors (NRTIs) and non-nucleoside reverse transcriptase inhibitors (NNRTIs). Recent phase III clinical trials have shown that two HIV-1 RT mutations, E138K and M184I, were the most frequent mutations found in patients that experienced virological failure during therapy that included rilpivirine (RPV), emtricitabine (FTC), and tenofovir (TDF).

## Methods

To investigate the mechanistic basis for resistance caused by the E138K and M184I mutations we used transient kinetics to characterize the enzymatic properties and drug susceptibility of RTs with these mutations and determined the biochemical mechanism of resistance to RPV. Specifically, we compared wild-type (WT) RT to RTs mutated in one or both of the enzymatic subunits: p66M184I/p51M184I, p66E138K/p51E138K, p66E138K/p51M184I, p66E138K/p51WT, p66WT/p51138K.

## Results

Our results show that M184I reduces the catalytic efficiency of RT by more than two-fold (p66M184I/p51M184Ihas more than 2-fold reduced kpol/Kd.dNTP with respect to WT). This defect is compensated by mutation E138K either in both subunits or only in p51 subunit (p66WT/p51138K).

## Conclusion

None of the mutations affected the template-primer binding affinity of RT. As expected, M184I does not reduce the susceptibility to RPV. Instead, RPV resistance is achieved by reduction in the binding affinity of the drug to RT because of the E138K mutation in the p51 subunit.

